# Impact of transition on quality of life in patients with congenital adrenal hyperplasia diagnosed during childhood

**DOI:** 10.1530/EC-17-0094

**Published:** 2017-07-18

**Authors:** Anne Bachelot, Magaly Vialon, Amandine Baptiste, Isabelle Tejedor, Caroline Elie, Michel Polak, Philippe Touraine, the CRMERC study group

**Affiliations:** 1 Department of Endocrinology and Reproductive Medicine and Centre de Référence des Maladies Endocriniennes Rares de la croissance et Centre des Pathologies gynécologiques RaresAP-HP, IE3M, Hôpital Pitié-Salpêtrière, ICAN, Paris, France; 2 UPMC UnivParis, France; 3 AP-HPClinical Research Unit Paris Descartes Necker Cochin, Paris, France; 4 Department of Pediatric EndocrinologyGynecology and Diabetology, Centre de Référence des Maladies Endocriniennes Rares de la Croissance et Centre des pathologies gynécologiques Rares, Hôpital Universitaire Necker Enfants malades, Paris, France; 5 Université Paris DescartesParis, France

**Keywords:** congenital adrenal hyperplasia, quality of life, transition, health care, 21OH deficiency, 21-hydroxylase

## Abstract

**Background:**

Health-related quality of life (QoL) in adult patients with congenital adrenal hyperplasia (CAH) has been variously reported. However, there is no study evaluating the impact of transition on quality of life.

**Methods:**

Adult patients with classic or non-classic CAH diagnosed during childhood CAH, born between 1970 and 1990, were recruited from the registers of Pediatric departments belonging to the French reference center for endocrine rare disease. Primary end point was the QoL (WHOQOL-BREF).

**Results:**

Seventy-three patients were included in the study, among them 59/73 were transferred to adult endocrinologist by their pediatricians for transition. WHOQOL-BREF scores were similar between patients with or without transition to specialist adult services, except for environment dimension score, which was slightly higher in CAH patients without transition. However, CAH patients with a regular follow-up had a better physical health, psychological health and environment score and item global QoL than the group without regular follow-up after transition.

**Conclusion:**

Regular medical follow-up in adulthood is associated with the transition between pediatric and adult care and is associated with better QoL in adults with CAH.

## Introduction

Transition is defined by the Society for Adolescent Medicine as a ‘purposeful, planned movement of adolescents and young adults with chronic physical and medical conditions from child-centered to adult-oriented health care system’ (1). Congenital adrenal hyperplasia (CAH) is a lifelong condition. CAH is classified according to symptoms, age of presentation and genetics and is usually divided into two forms: the classic or severe form and the non-classic form (2). Pediatric endocrine care is delivered through specialist services, and the recommendation is that patients with CAH should remain within specialist services as adults. Management of adolescents with congenital adrenal hyperplasia presents unique challenges (3, 4). Once growth is no longer a concern, a shift in treatment goals from optimization of growth and puberty to prevention of long-term adverse outcomes and optimization of fertility and sexual function is needed (5, 6, 7, 8, 9, 10).

The organization of transition is important in order to support and encourage these patients in this stressful period. The goal is also not to lose follow-up. However, very few studies have examined transitional care and its determinants in real life. Transition of care is a time when many patients with CAH stop adherence to their medication (3, 4, 11). A German study insists on the lack of support concerning psychosexual development during transition period (4). Unfortunately, many patients in adulthood are lost to follow-up. An audit of transition of CAH patients in the United Kingdom showed that 50% of patients with CAH had poor biochemical control and/or adverse clinical consequences, and 50% who were transferred to specialist adult services were lost to follow-up (12).

Health-related quality of life (QoL) in adult patients with CAH has been variously reported (13, 14, 15). The reason for this heterogeneity in QoL reporting for CAH adults is debated but could relate to variables including treatment regimen, health care provision in different countries and genetics.

As there is no study evaluating the impact of transition on QoL and on the health status in CAH patients, we designed a study to evaluate the impact of a transition process and of a regular medical follow-up on QoL and on health status in a cohort of patients with classic and non-classic CAH diagnosed during childhood.

## Patients and methods

### Patients

Patients were recruited from the registers of Pediatric departments belonging to the French reference center for endocrine rare disease (CRMERC), including 3 academic department of pediatric endocrinology located in Paris: Necker Hospital, Trousseau Hospital and Robert Debré Hospital. Patients were also recruited from the registers of the adult department of Endocrinology and Reproductive Medicine located in Pitié Salpêtrière Hospital. Ethical approval was granted by the Ethics Committee of Pitié-Salpêtrière Hospital (Paris, France). Consent has been obtained from each patient or subject after full explanation of the purpose and nature of all procedures used.

Inclusion criteria were adult patients born between 1970 and 1990; presence of classic or non-classic CAH diagnosed during childhood (<10 years), according to the predicted severity of the mutations. Primary end point of the study was to compare the quality of life of adult CAH patients transitioned or not from pediatric care to specialist adult services. Secondary end points were comparison of adult height and difference compared to target height, body mass index (BMI), menstrual cycle regularity in women and plasma 17OH-progesterone and renin concentrations in adult CAH patients with or without transition.

All patients eligible to enter this study were contacted by mail, e-mail or phone. In the event that no response was received, reminders were sent once, 3 months later. Again, in the event that no response was received, the general practitioner of the patient was contacted and recent address e-mail or phone was then used to contact the patient. In the absence of answer after this procedure, a letter with information notice of the study and WHOQOL was sent to the patient. Transition was defined by the transfer from pediatric care to specialist adult services, associated with the tailored transition program of each department. Regular follow-up was defined by the presence of 1 or more visits per year with an endocrinologist and without interruption period in this follow-up. All others were considered as not having gone through transition successfully.

### Study design

All patients were seen early in the morning on the day of the visit for the study, and standard anthropometric measurements were obtained. BMI was calculated as weight/(height)^2^ (kg/m^2^). Data concerning presentation, diagnosis, medical treatment, conduct of a transition or not and other relevant information were reviewed. Regularity of menses was evaluated in women before the beginning of any therapy known to interfere with the menstrual cycle. The patients were divided into classic salt-wasting (SW) form, classic simple virilizing form (SV) and non-classic form according to the clinical data and the predicted severity of the mutations. All subjects fasted for at least 9 h before sampling. Blood samples for the measurement plasma 17OH-progesterone were taken 1–2 h after administration of the morning medication. Samples were centrifuged and separated immediately after collection and were stored at 20°C until assayed.

### Quality of life

The data collection instrument was the World Health Organization Quality of Life (WHOQOL)-BREF questionnaire, which is the summarized form of the comprehensive 100-question QoL measurement. It consists of 26 questions, scored on a 5-point Likert scale, which monitor different aspects of an individual’s QoL. In this questionnaire, two questions were related to the patients’ general feelings about their QoL. The remaining questions were related to the patients’ feelings and behaviors during the previous 2 weeks in the physical dimension (physical activities, drug dependency and supportive medicines, mobility, pain and feeling of discomfort, sleep and rest and the ability to perform activities), psychological dimension (feeling toward body posture and appearance, positive and negative feelings, learning, thoughts, memory and concentration, self-confidence and personality traits), social dimension (personal relationships, social support) and environmental dimension (financial sources, freedom and physical security, accessibility to social and health care, house condition, accessibility to new data and various skills, opportunity to take part in social activities and physical environment such as pollution, noise, traffic and transportation). One question was related to the sexual dimension. Every question has a score range from 0 to 4; 0 represented the worst and 4 represented the best conditions of QoL. Achieved scores for each dimension were converted into a standardized form, ranging from 0 to 100, following scoring instructions described in the manual of the WHO QoL-BREF questionnaire. This questionnaire has been validated in the French general population (16, 17).

### Statistical analysis

All statistical analyses were performed using R software, version 2.11.1. A descriptive analysis of all patients was conducted. Results were reported as mean ± s.d. (in case of normal distribution), or median (min; max) (in case of non-normal distribution) for continuous variables and as frequency counts and percentages (%) for categorical variables. Characteristics of patients agreeing to participate (and thus included) in the study were first compared to those of patients who expressed a refusal (or did not answered) or were lost to follow-up.

A comparison was then performed among included patients according to whether they had a successful transition or not. In a subsequent exploratory analysis, we further examined the differences between patients with or without regular follow-up after their organized transition.

Chi-square or Fisher’s exact tests were used to test for the association between categorical variables depending on expected frequencies. Differences in the distribution of continuous outcomes between two groups were investigated using Student *t*-tests or non-parametric Wilcoxon signed-rank tests (in case of non-normal distributions) were computed. For more than two groups, ANOVA tests or non-parametric Kruskal–Wallis tests were computed.

All tests used a two-sided significance level of 0.05.

## Results

### Population

**Figure 1:**
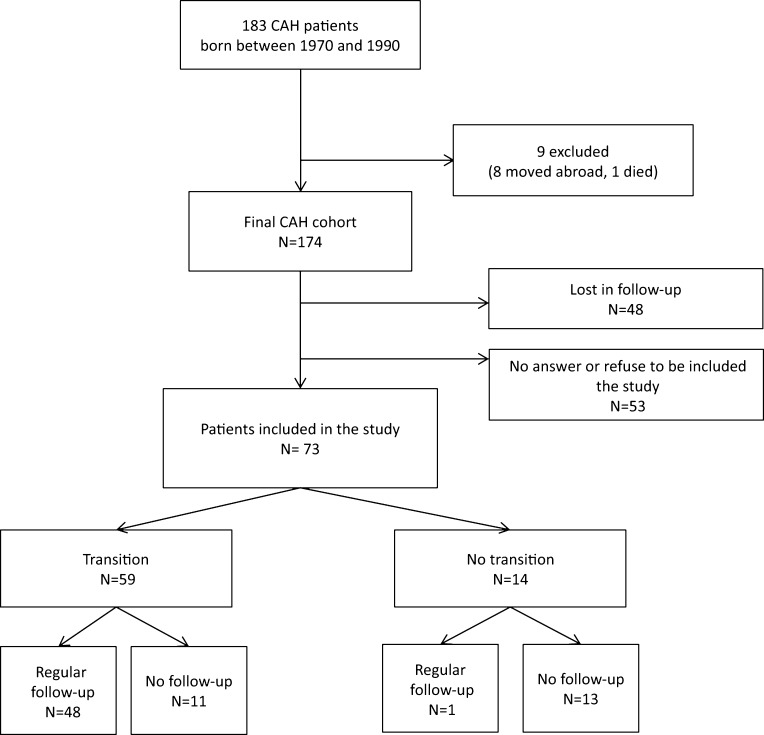
CAH patients included in the study.

One hundred and eighty-three patients were eligible to participate in this study, 9 were excluded (8 had move abroad, 1 died (not related to CAH)). The CAH cohort was therefore 174 patients. Seventy-three patients agreed to take part in the study (42%) (Fig. 1). The remaining 101 patients were either lost to follow-up (*n* = 48, 28%) or declined the study or did not answer (*n* = 53, 30%) (Fig. 1). These 101 patients did not differ from the 73 studied patients in term of clinical or hormonal presentation, except for the age at the end of the study (34.8 ± 5.5 years for the group lost to follow-up, 31.7 ± 5.7 years for the group who refuse to participate and 32.7 ± 6.2 years for patients included, *P* = 0.02) (Table 1). Patients included in the study were less frequently salt-wasters (48 vs 75% for the group lost to follow-up and 61% for the group who refuse to participate). Transition program was proposed to 80% of the patients included in the study, 35% of the patients lost to follow-up and 64% of the patients, which refuse to participate (Table 1).

**Table 1:** Clinical characteristics of the CAH patients eligible to the study.

	Included (*n* = 73)	Lost to follow-up (*n* = 48)	Refuse to participate (*n* = 53)	*P*
Gender				
Women	48	34	34	0.76
Form of CAH				0.09
Classic				
Simple virilizing form	15	4	6	
Salt-wasting	35	27	30	
Non-classic	23	5	13	
Age at diagnosis	0.5 (0.0; 14.0)	0.00 (0.0; 8.0)	0.00 (0.0; 9.0)	0.09
Prader classification (women)				0.07
Normal	19	3	6	
I ou II	6	4	3	
III ou IV	18	16	17	
Finale height (cm)				
Women	157.0 ± 6.8	154.0 ± 8.7	159.2 ± 6.0	0.12
Men	168.4 ± 6.6	171.6 ± 6.7	168.9 ± 7.1	0.64
Age at the end of the study (years)	33 (24; 44)	34 (25; 44)	30 (24; 43)	0.02
Organized transition process	59	17	34	0.30

Among the 73 patients included in the study, 59 were transferred to specialist adult services by their pediatricians for transition. Among them, 48/59 had a regular medical follow-up in adulthood (Fig. 1). Among the 14 patients not transferred to specialist adult services, only one had a regular medical follow-up in adulthood (Fig. 1).

### QoL results

CAH patients were compared regarding the existence or not of a transfer to adult specialist services (patients with or without transition, Table 2). There was less women in the group with transition, even though it did not reach the significant level. Patients with transition were younger than the other group (*P* = 0.02) and were more frequently presenting SW CAH (32/59 patients with transition vs 3/14 without transition, *P* = 0.03) (Table 2). WHOQOL-BREF scores were generally similar between groups, except for environment dimension score, which was slightly higher in CAH patients without transition (*P* = 0.01, Table 2).

**Table 2:** Clinical characteristics of the CAH patients and results of WHOQOL-BREF according to transition

	Patients with transition (*n* = 59)	Patients without transition (*n* = 14)	***P***
Gender			
Women	36	12	0.12
Form of CAH			0.03
Classic			
Simple virilizing form	9	6	
Salt wasting	32	3	
Non-classic	18	5	
Age at diagnosis (years)	0.0 (0; 14)	1.0 (0; 10)	0.70
Age at inclusion (years)	30.0 (23; 43)	36.0 (25; 43)	0.02
Way of life			0.11
Single	15	7	
In couple	26	5	
Live with parents	18	2	
WHOQOL-BREF			
Dimensions			
Physical health	67.9 (39.3; 85.7)	71.4 (50.0; 89.3)	0.11
Psychological health	62.5 (16.7; 83.3)	60.40 (29.2; 83.3)	0.66
Social relationships	75.0 (8.3; 100.0)	70.8 (8.3; 100.0)	0.87
Environment	75.0 (28.1; 100.0)	79.6 (68.8; 96.9)	0.01
Items			
Global quality of life	4.0 (2.0; 5.0)	4.00 (2.0; 5.0)	0.22
Global health	4.0 (1.0; 5.0)	4.0 (2.0; 5.0)	0.26

Successful transition was defined by regular follow-up of patients after the transfer to adult specialist services. This allowed us to analyze not only the impact of a transition but also the real impact of a successful transition on the QoL and on clinical and hormonal parameters (Table 3). The only difference between the two groups was the repartition of gender: less women were lost to follow-up after transition process (33/36 women had a regular follow-up vs 15/23 men, *P* = 0.02). CAH patients with a successful transition had a better physical health, psychological health and environment score and item global QoL than the group without regular follow-up after transition (Table 3). There was no difference between the two groups regarding patient’s level of education. Nevertheless, regarding socioeconomic status, patients with regular follow-up were more frequently managerial (10/48 vs 1/11) or intermediate (10/48% vs 0/11). Unfortunately, multivariate analysis or adjusted results could not be performed due to the low sample size.

**Table 3:** Clinical characteristics and results of WHOQOL-BREF of CAH patients with or without successful transition.

	Regular follow-up after transition (*n* = 48)	**No regular follow-up after transition** (*n* = 11)	***P***
Gender			0.02
Women	33	3	
Form of CAH			0.53
Classic			
Simple virilizing form	8	1	
Salt-wasting	24	8	
Non-classic	16	2	
Age at inclusion (years)	30.0 (23.0; 43.0)	33.0 (23.0; 43.0)	0.66
WHOQOL-BREF			
Dimensions			
Physical health	69.6 (42.9; 85.7)	57.0 (39.3; 75.0)	0.03
Psychological health	65.0 (41.7; 83.3)	50.0 (16.7; 64.6)	0.01
Social relationships	75.0 (8.3; 100.0)	66.7 (8.3; 91.7)	0.11
Environment	78.1 (34.4; 100.0)	62.5 (28.1; 84.4)	0.04
Items			
Global quality of life	4.0 (3.0; 5.0)	4.0 (2.0; 5.0)	0.02
Global health	4.0 (2.0; 5.0)	4.0 (1.0; 5.0)	0.21

### Secondary end points

Secondary end points were only available for 49 patients. Patients with transition (*n* = 39, among them 32 with regular follow-up) were compared to patients without transition (*n* = 10, none with regular follow-up). There were 25 women among the 39 patients with transition, and 9 women among the 10 patients without transition (*P* = 0.14). Adult height was the same between the women in the two groups (1.57 m (1.45; 1.70) vs 1.57 m (1.38; 1.67), *P* = 0.68). BMI did not differ between the two groups (25.1 kg/m² (18.3; 47.6) vs 25.1 kg/m² (21; 38.5), *P* = 0.74). Menstrual cycles were regular for around two thirds of the patients in each group. There was no difference between 17OH-progesterone and renin concentrations between the two groups (12.8 (0.8; 193.0) ng/mL vs 8.9 (0.5; 133) ng/mL, *P* = 0.32 and 19.0 pg/mL (0.3; 248.7) vs 14.3 pg/mL (6.4; 61.9), *P* = 0.32 respectively).

## Discussion

Much emphasis has recently been placed on the importance of transitional care for chronic nonendocrine and endocrine diseases in childhood; however, very few studies have examined transitional care and its determinants (18). Improving transition from pediatric to adult endocrine care is a recognized challenge. Although there is emerging evidence about how to organize transition, there is a need to identify which patients are at risk of drifting away from endocrine care and to evaluate the impact of a successful transition (12). A recent study in the United Kingdom, however, estimated the numbers of patients with CAH attending specialist adult services between 2 and 5%. Inadequate transition to adult services was emphasized as a potential explanation (7). An assessment of a single centre’s experience of transitioning patients with CAH to specialist adult services has highlighted that the difficulty of a good health status at transfer and engagement with adult services (12). In this audit, 50% of patients with CAH had poor biochemical control and/or adverse clinical consequences, and 50% who were transferred to specialist adult services were lost to follow-up (12). Introduction of young person clinic increased the numbers of patients being transferred to specialist adult services but failed to improve engagement (12). In our study, we confirmed these data, as almost a third of the CAH patients were lost to follow-up in adulthood, despite a regular care in tertiary medical center during childhood. We also demonstrated that transition was closely linked to regular medical care during adulthood. It reinforces the concept of a well-organized transition (3, 7, 12). Indeed, in this paper, transition was defined by the transfer from pediatric care to specialist adult services, associated with the tailored transition program of each department, and these programs are in constant evolution and evaluation (19).

Health-related quality of life (QoL) in adult patients with CAH has been variously reported (13, 14, 15, 20, 21, 22). Three groups have reported better, similar or mildly impaired QoL in CAH patients compared with controls or the normal population (14, 15, 20), while other publications have reported poor QoL in CAH adults (13, 21, 22, 23, 24). The reason for this heterogeneity in QoL reporting for CAH adults is still debated but could relate to variables including treatment regimen, health care provision in different countries and genetics, but seems not related to genotype (severity of the mutations) or phenotype (classic or non-classic). Our study on a large cohort of CAH adults is the first to report on the impact of transition and specialist medical care on QoL. We found that global QoL was not different in patients with or without transition but was better in adult CAH patients with regular medical follow-up and a successful transition. We had previously reported on the evaluation of the transition process and on the needs expressed by patients with chronic endocrine conditions at transition (19). This earlier pilot study involving 13 CAH patients has shown that the vast majority of subjects had a good knowledge of their medication. Patients were also asked at the initial visits whether specific medical concerns about adult CAH had been properly addressed. Most women (67%) and all men were satisfied with the information received about fertility and genetic transmission to offspring (19).

Limitations in this study include its cross-sectional nature, the loss to follow-up and the large number of variables examined. Indeed, as 101/174 patients were not included in the study, we have no information about most of the patients, which is a general problem with studies of CAH adults. We cannot fully exclude that the 14 subjects who did not undergo successful transition belong to a special group of patients prone to decreased QoL and reluctant to undergo adult follow-up in a specialized adult department. Moreover, patients with regular follow-up belonged to a higher socioeconomic class, which could impact on the results of QoL; however, due to the low sample size, this hypothesis could not be tested, as multivariate analysis or adjusted results could not be performed. Future prospective studies designed specifically to examine the long-term effects of a successful transition CAH patients are needed to address the limitations of this study.

Impact of a successful transition on future health status is not known. In this study, we could not highlight a difference between the different groups regarding comorbidity described in CAH patients, i.e. BMI, and reproductive function in women. This could be explained by the reduced number of patients included in this secondary analysis, as the study and the number of patients included were not designed to demonstrate it. Longitudinal studies have to answer more precisely these questions.

In conclusion, regular medical follow-up in adulthood is closely related to the transition between pediatric and adult care and is associated with better QoL in adults with CAH. The ideal conditions of pediatric to adult care transition, associated with training, counseling and education and the medical consequences of a successful transition have to be further studied.

## Declaration of interest

The authors declare that there is no conflict of interest that could be perceived as prejudicing the impartiality of the research reported.

## Funding

Funding for this study was provided by a grant from the Pfizer Foundation.
